# Germany floods—a warning for future extreme weather events

**DOI:** 10.1016/j.lanepe.2021.100194

**Published:** 2021-08-02

Parts of western Europe, including Germany, Belgium, and the Netherlands, have witnessed the worst flooding Europe has seen in living memory, after record rainfall caused swollen rivers to burst their banks beginning on July 13, 2021. With over 200 deaths as of July 22 (more than 170 in Germany and 32 in Belgium), hundreds injured, and many more still unaccounted for, Germany has been hit the hardest by this once in a century flooding event. The German states of Rhineland-Palatinate and North Rhine-Westphalia and the Belgian city of Liège suffered the highest number of causalities. Fast-moving flood waters tossed and swept away vehicles, submerged villages, triggered landslides, destroyed water supply and discharge lines, and left over 200 000 homes without power. Members of the German military were deployed to help with rescue efforts, assisted by flood rescue teams from France, Italy, and Austria. The EU activated its Civil Protection Mechanism to support local flood rescue teams and the German government has announced over €300 million in aid, though the damage is expected to be in billions of euros.

These floods do make it clear that much of Europe is ill equipped to deal with heavy rains. In Germany, the breakdown of the early warning system and the failed response to evacuate people in advance, begs for accountability. There was a communication breakdown between officials, media, and the public in many affected areas. As a result, local authorities were ill prepared, and people took fewer precautions. The head of Germany's Federal Office of Civil Protection and Disaster Assistance (BBK), Armin Schuster, rejected criticism that more needed to be done to improve the country's warning system for extreme weather. He has maintained that 150 warnings were issued and that the warning infrastructure was not to blame, but rather how local authorities and communities respond to these warnings. The Weather agency, German Meteorological Service (DWD), also defended itself, saying that the agency acted according to its mandate—to warn local authorities of expected weather patterns—but regrettably, these messages were not passed on to the communities; moreover, in Germany, local districts are responsible for risk management, and not the DWD.

While researchers are discussing the multifactorial climatic, hydrological, and societal factors that caused the flooding, the knee-jerk response by the German government has been to blame it on climate change. It is challenging to attribute individual flooding /weather events to climate change, but there is a clear link between global heating and extreme weather events. With every 1°C rise in temperature, the air can take up more moisture (about 7% per degree Celsius rise in temperature) and hold it for longer, leading to more intense drought and also more intense rainfall once it is released—a pattern of extreme weather that is expected to continue worsening with further warming. In addition to climate change, there are several landscape factors that contribute to flooding, including soil saturation, topography, and urban development, which increases the rate of runoff. Wider land use can also be a contributing factor: for example, the river Rhine (the second-longest river in central and western Europe) has already lost four-fifths of its natural flood plains, and urbanisation can also increase the risk of rapid runoff and flooding.

This scale of flooding catastrophe has enhanced the focus on climate change in Germany and could shake up the course of Germany's general election in September 2021. Leading political parties are putting more emphasis on measures for climate protection and promising tougher policies to help cut CO_2_ emissions. Along with these long-term goals, concrete steps for future adaptation from government and society are needed, including making homes flood- and windproof, infrastructure planning, public awareness, diversification of land use, and economic planning at the national level to deal with multiple aspects of sustainability simultaneously. A firm commitment is needed to mitigate and tackle emissions and to fulfil the Paris agreement—to limit global warming to well below 2°C, preferably 1·5°C, compared with pre-industrial levels. Coincidentally, in July, the European Commission unveiled its most ambitious plan yet to tackle climate change, the "Fit for 55", a package of revisions and regulations targeted at drastically cutting greenhouse gas emissions—a plan to reach a 55% reduction in carbon emissions from 1990 levels by 2030 (the current target was to reduce carbon emissions from cars by 37•5% from 1990 levels by 2030). The overarching goal is to make the EU carbon neutral by 2050, as part of the European Green Deal. The proposals still need to be approved by the bloc's 27 member states and the European Parliament.

Climate change is known to contribute to the intensification of extreme weather events and almost certainly played a part in the extreme rains and subsequent floods in Europe. These events reveal that no one is immune to the kinds of weather events that are being exacerbated under climate change. Consequently, any steps taken to mitigate climate change will help to avoid worsening weather extremes in the future. However, governments and citizens also need to do better with respect to being ready and prepared for these extreme weather events already occurring. Germany´s devastating floods is a clear warning of the dangers low-lying cities across the world will face, as climate change will escalate extreme weather.

*The Lancet Regional Health – Europe*

